# Effect of isolated lactic acid bacteria on the quality and bacterial diversity of native grass silage

**DOI:** 10.3389/fpls.2023.1160369

**Published:** 2023-07-06

**Authors:** Jian Bao, Gentu Ge, Zhijun Wang, Yanzi Xiao, Muqier Zhao, Lin Sun, Yu Wang, Jiawei Zhang, Yushan Jia, Shuai Du

**Affiliations:** ^1^ Key Laboratory of Forage Cultivation, Processing and High Efficient Utilization of Ministry of Agriculture and Rural Affairs, Key Laboratory of Grassland Resources of the Ministry of Education, College of Grassland, Resources and Environment, Inner Mongolia Agricultural University, Hohhot, China; ^2^ College of Agriculture and Forestry, Hulunbuir University, Hulunbuir, China; ^3^ Inner Mongolia Academy of Agricultural and Animal Husbandry Sciences, Grassland Research Institute, Hohhot, China

**Keywords:** native grass, steppe types, lactic acid bacteria, silage quality, bacterial community

## Abstract

**Objective:**

The objective of this study was to isolate lactic acid bacteria (LAB) from native grasses and naturally fermented silages, determine their identity, and assess their effects on silage quality and bacterial communities of the native grasses of three steppe types fermented for 60 days.

**Methods:**

Among the 58 isolated LAB strains, *Limosilactobacillus fermentum* (BL1) and *Latilactobacillus graminis* (BL5) were identified using 16S rRNA sequences. Both strains showed normal growth at 15- 45°C temperature, 3-6.5% NaCl concentration, and pH 4-9. Two isolated LAB strains (labeled L1 and L5) and two commercial additives (*Lactiplantibacillus plantarum* and *Lentilactobacillus buchneri*; designated as LP and LB, respectively) were added individually to native grasses of three steppe types (meadow steppe, MS; typical steppe, TS; desert steppe, DS), and measured after 60 d of fermentation. The fresh material (FM) of different steppe types was treated with LAB (1 × 10^5^ colony forming units/g fresh weight) or distilled water (control treatment [CK]).

**Results:**

Compared with CK, the LAB treatment showed favorable effects on all three steppe types, i.e., reduced pH and increased water-soluble carbohydrate content, by modulating the microbiota. The lowest pH was found in the L5 treatment of three steppe types, at the same time, the markedly (*p* < 0.05) elevated acetic acid (AA) concentration was detected in the L1 and LB treatment. The composition of bacterial community in native grass silage shifted from *Pantoea agglomerans* and *Rosenbergiella nectarea* to *Lentilactobacillus buchneri* at the species level. The abundance of *Lentilactobacillus buchneri* and *Lactiplantibacillus plantarum* increased significantly in L1, L5, LP, and LB treatments, respectively, compared with CK (*p* < 0.05).

**Conclusion:**

In summary, the addition of LAB led to the shifted of microbiota and modified the quality of silage, and *L. fermentum* and *L. graminis* improved the performance of native grass silage.

## Introduction

Meadow steppe, typical steppe, and desert steppe are three common steppe types in northern temperate Asia, with relatively taste ability and the capacity to meet livestock growth (Hou et al., 2017). Moreover, these steppe types are tolerant to cold, drought, sand, and biotic stresses (Pang et al., 2011; Yuan et al., 2012; Fu et al., 2014), and play a key role in meeting the needs of animal production and, indirectly, protecting human health (Wang et al., 2022). Traditionally, these steppes have been commonly used to make hay or as pasture for grazing ruminants (Zhang et al., 2018). Generally, native grass hay is the traditional method of native grass; however, because the quality and palatability of natural hay is sensitive to environmental factors, it is not feasible to address the seasonal and annual imbalances through hay preparation techniques (Li et al., 2015). Ensiling, a traditional method of preserving animal feed and fresh fodder crops, provides feed for animals all year round, effectively reducing nutritional losses and extending storage time (Wang et al., 2022). Silage was mainly lactic acid bacteria (LAB) in the anaerobic environment using soluble carbohydrates for fermentation, produce lactic acid (LA), leading to low pH, inhibit aerobic bacteria, effective preservation of nutrients and improves feed palatability (Cai et al., 2020).

Our previous study showed that obtaining high-quality silage from different native grass steppes through direct fermentation is difficult (You et al., 2021). The addition of LAB is an effective way to overcome the poor fermentation characteristics of native grasses (Jaipolsaen et al., 2021). Additionally, LAB can inhibit the proliferation of harmful microorganisms and reduce the pH value, improving the quality of silage (Xian et al., 2022). Nevertheless, one of the decisive elements for the successful LAB inoculation of forage silage is the good positive interaction between microorganisms and raw materials (Avila et al., 2014). Previous studies showed that the effect of LAB obtained from stylo, oat, and sweet sorghum is comparable with or even better than that of commercial bacteria (Liu et al., 2012; Sifeeldein et al., 2019; Li et al., 2021). These studies suggest that the forage crop is the best source of suitable isolates. Thus, the selection and application of LAB for making different types of silage is essential, but few LAB have been isolated from different the native grasslands of the Mongolian Plateau. The complex and diverse geographical environment and climatic conditions of the Mongolian Plateau have bred rich and unique LAB resources.

In recent years, the third-generation Pacific Biosciences (PacBio) single-molecule real-time (SMRT) DNA sequencing technology has improved classification accuracy and speed by producing ultra-long reads, directly detecting epi-modification sites and avoiding template amplification (Xu et al., 2018). The SMRT technology can identify organisms to the species level and has been applied to the silages of Italian ryegrass and stylo (Wang et al., 2019; Yan et al., 2019). Consequently, the effects of different LAB on changes in the microbial composition of silage could be evaluated more accurately. Additionally, the effect of LAB obtained from natural sources on the diversity patterns of bacterial communities of native grass silage has been evaluated using the PacBio SMRT method in different steppe types.

In our study, we selected two high-potential strains, *Limosilactobacillus fermentum* and *Latilactobacillus graminis*, isolated from native grasses of the Mongolian Plateau. These two strains use multiple carbohydrates, produce more LA and acetic acid (AA) in an anaerobic environment and grow under low pH conditions; however, how these strains change the fermentation quality of different native grass steppes remains unclear. We hypothesized that LAB isolated from their own material might be better for improving the quality of three steppe types native grass silage relative to commercial LAB. In this study, we aimed to 1) determine the effects of different LAB on the fermentation characteristics of three steppe types of native grass silage; 2) evaluate the changes in bacterial communities and their functional properties; and 3) investigate the priority effects and mechanisms of action of *L. fermentum* and *L. graminis* on silage bacterial communities.

## Materials and methods

### LAB strains

A total of 58 LAB strains were isolated from 48 samples of native grass and naturally fermented silage, according to the method of Cai et al. (1998). Two representative strains of LAB(BL1 and BL5) were selected from 58 isolates based on their morphological and physiological tests, growth curve tests, and acid production curve tests. Native grasses in the meadow steppe (MS), typical steppe (TS), and desert steppe (DS) of the Inner Mongolia Plateau in China were harvested at the milk stage in July 2019 and 2020. The following species were predominant in the three steppes: Baical Needlegrass (*Stipa baicalensis* Roshev.) and Chinese Leymus (*Leymus chinensi*s [Trin.] Tzvel.) in MS; Larch Needlegrass (*Stipa grandis* P. Smirn.) and Chinese Leymus (*L. chinensis* [Trin.] Tzvel.) in TS; and Short-flowered Needlegrass (*Stipa breviflora* Griseb.) and Mongolian Leek (*Allium mongolicum* Regel.) in DS. Each sample (10 g) was mixed with 90 ml of sterile distilled water. The dilutions were spread on Deman, Rugose, Sharp (MRS) agar (Difco Laboratories, Detroit, MI, USA), and incubated under anaerobic conditions at 35°C for 48 h to isolate LAB. In addition, putative homogeneous and heterogeneous LAB strains were stored in MRS containing 25% glycerol at -80°C by strain purification on MRS agar plates.

### Physiological and morphological tests

Gram staining, catalase reaction, colony morphology, and glucose-based gas production tests were performed as described by Kozaki et al. (1992). Temperature, acid and salt tolerance tests of the strains were performed using MRS broth (Cai et al., 1999). Carbohydrate assimilation of the strains was determined using API 50 CH (BioMerieux, Marcy l’Etoile, France) (Zhang et al., 2015).

### Identification of LAB strains by 16S rRNA sequence analysis

Genomic DNA of the screened strains was extracted using the Bacterial DNA Kit (Tiangen Biotech Co., Ltd., Beijing, China). The isolated DNA was subjected to polymerase chain reaction (PCR) using primers 27F (5’-AGAGTTTGATCCTGGCTCAG-3’) and 1492R (5’-TACGGCTACCTTGTTACGACT-3’) (Tohno et al., 2012; Pitiwittayakul et al., 2021). Then, 16S rRNA sequences were identified using BLAST analysis in the GenBank database (Ennahar et al., 2003).

### Preparation of silage

Native grasses in MS (119°13′E, 44°04′N), TS (116°28′E, 44°05′N), and DS (113°34′42″E, 44°6′N) of the Inner Mongolia Plateau, China, were harvested at the milk stage in July 2021. A sample square (50 m × 100 m) divided into five paired quadrants (100 cm × 100 cm) was selected and used to collect fresh forage samples from the three steppe types. Freshly harvested material was cut into 20-30-mm long pieces using a manual forage chopper (Mode-8, 200; Minghong Business, Shandong, China), placed in polythene bags, and vacuum sealed. Two screened LAB strains (BL1 and BL5; designated as L1 and L5, respectively) and two commercial LAB additives (*Lactiplantibacillus plantarum* and *Lentilactobacillus buchneri*; designated as LP and LB, respectively (Shandong Zhongke Jiayi Biological Engineering Co. Ltd., China) were added individually at a concentration of 10^5^ cfu/g of fresh material (FM), with distilled water as a control (CK). The FM and additives were mixed thoroughly, and silage (approximately 250 g) was packed in polyethylene bags (Shenyang Huasheng Plastic Packaging Products Co., Ltd., China). The bags were then sealed with a vacuum sealer to extract the air. Three replicates were made for each treatment, and the silage of different treatments were measured after 60 days of ensiling at room temperature (25°C).

### Analyses of chemical composition, microorganism and fermentation parameter

Three replicates were set up for each native grass sample. Dry matter (DM) and content crude protein (CP) were measured following the method of Zhang et al. (2016) and Du et al. (2019). Determination of neutral detergent fiber (NDF) and acid detergent fiber (ADF) content was performed according to the method of Van Soest et al. (1991) by using the Ankom A2000i fiber analyzer (Ankom Technology, Macedon, NY, USA). The water soluble carbohydrates (WSC) content was measured following the method of Chen et al. (2015).

A sample of silage (10 g) was mixed with 90 g deionized water following the description of Yuan et al. (2020), and stored in a refrigerator at 4°C for 24 h. The leachate was filtered through four layers of gauze and filter paper, with measurements of pH, ammonia nitrogen (NH_3_-N) and organic acids in the leachate. pH was measured using a glass electrode pH meter (Leici pH S-3C, Shanghai, China). The content of LA, AA, propionic acid (PA) and butyric acid (BA) in silages was determined by high performance liquid chromatography (HPLC; model: Waters e2695, Milford, USA) (Cheng et al., 2020). The method of Broderick and Kang (1980) was used to determine NH_3_-N concentrations. Microbial populations (LAB, yeasts, mold, anerobic bacteria, and coliform bacteria) in the FM were assessed as described in a previous report (Guo et al., 2021a).

### DNA extraction, PCR and sequencing

The bacterial community composition of native grass fermented for 60 days was analyzed by 16S rRNA gene sequencing. Based on the investigation of Liu et al. (2019), total DNA was extracted from fresh and silage samples of native grass. The metagenomic DNA extraction and PCR amplification procedures of bacterial 16S ribosomal RNA genes were performed as per Guo et al. (2021).

DNA was amplified with primers 27F (5’-AGRGTTTGATYNTGGCTCAG-3’) and 1492R (5’-TASGGHTACCTTGTTASGACTT-3’). PCR conditions were an initial denaturation at 98°C for 2 min, 30 cycles of denaturation at 98°C for 30 s, annealing at 50°C for 30 s, and elongation at 72°C for 60 s, followed by a final extension at 72°C for 5 min. The PCR products were purified for sequencing and analysis. Each treatment was performed in triplicate. 16S rRNA gene sequence were stored in NCBI with BioProject accession number PRJNA912573.

NGS sequencing was performed by Biomarker Technologies (Beijing, China) on a Pacbio_SMRT platform (Pacbio Sequel II, CA, USA). Coverage of α-diversity indicators Chao1 and Good was calculated using QIIME v1.9.1 (Segata et al., 2011). Principal coordinate analysis (PCoA) was performed using the R program (version 3.2.5) on the basis of β-diversity unweighted or weighted unifrac distances. Operational taxonomic units (OTUs) were classified using Ribosome Database Project (RDP) Classifier (version 2.2) against the SILVA (Release 128) 16S rRNA database with a minimum confidence cut off of 0.7, and were then denominated at the phylum, genus, and species levels. Mothur (version v.1.30) software was used to evaluate the α-diversity indices (ACE, Chao 1, Simpson, and Shannon) of the samples. LEfSe (Linear Discriminant Analysis (LDA) effect size) was able to find biomarkers that are statistically different between groups. It was performed using a free online platform (https://international.biocloud.net). Phylogenetic Investigation of Communities by Reconstruction of Unobserved States II (PICRUSt2) software was used to predict microbial functions from the Kyoto Encyclopedia of Genes and Genomes (KEGG) database.

### Statistical analysis

The effect of steppe types and ensiling time on silage quality was evaluated by two-way ANOVA and Duncan’s multiple range test. All statistical procedures were performed using SAS 9.3 software (SAS Institute, Inc., Cary, NC, USA). Effects were considered significant when *p* < 0.05. Figures for microbiota data were performed using the BMK Cloud platform online and GraphPad prism 8.

## Results

### LAB strain characteristics

Two isolated LAB strains were Gram-positive and catalase negative (**Table 1**). The strain BL5 was a homo-fermenter, while the strain BL1 was a hetero-fermenter. Additionally, BL1 showed optimal growth at 15-50°C and pH 3.5-9.0 but weak growth at 10°C and pH 3, whereas strain BL5 showed normal growth at 10-45°C and pH 4.0-9.0 but weak growth at 5°C and pH 3.5. The growth of strains BL1 and BL5 was normal at NaCl concentrations of 3.0% and 6.5%, respectively.

**Table 1:** he selection of isolated lactic acid bacteria on the base of the morphological and physiological tests.

Items	BL1	BL5
Shape	Rod	Rod
Gram stain	+	+
Gas for glucose	+	–
Catalase	–	–
Fermentation type	He	Ho
**Growth at temperature ( °C)**		
5.0	–	w
10	W	+
15	+	+
30	+	+
45	+	+
50	+	w
**Growth at Ph**		
3.0	W	–
3.5	+	w
4.0	+	+
4.5	+	+
8.5	+	+
9.0	+	+
**Growth in NaCl (%)**		
3.0	+	+
6.5	+	+

Ho, homo-fermentation; He, hetero-fermentation; w, weak; +, positive; -, negative.

Both strains exhibited different fermentation patterns, based on the results of gas detection on glucose. The two strains differ in the products of fermentation of carbohydrates such as D-Xylose, D-Mannitol, N-Acetyl Glucosamine, Amygdalin, Esculin, Salicin, Maltose, Lactose, Melibiose, Sucrose, Raffinose, β-Gentiobiose, and Gluconate (**Table 2**).

**Table 2:** The characteristics of isolated lactic acid bacteria on the base of carbohydrate fermentation.

Items	BL1	BL5
L-Arabinose	+	+
Ribose	+	+
D-Xylose	+	–
D-Galactose	+	+
D-Glucose	+	+
D-Fructose	+	+
D-Mannose	+	+
D-Mannitol	W	–
D-Sorbitol	–	–
Methyl-αD-Mannopyranoside	–	–
N-Acetyl Glucosamine	–	+
Amygdalin	–	w
Arbutin	–	–
Esculin	–	+
Salicin	–	w
Cellobiose	+	+
Maltose	+	–
Lactose	+	–
Melibiose	+	–
Sucrose	+	–
Trehalose	+	+
Melezitose	–	–
Raffinose	+	–
β-Gentiobiose	–	w
D-Tagatose	–	–
D-Arabitol	–	–
Gluconate	W	–

w, weak; +, positive; -, negative

The results of 16S rRNA sequencing were analyzed by BLAST in the GenBank database (**Table 3**). Strain BL1 showed high similarity to *Limosilactobacillus fermentum* (99.93%), and strain BL5 expressed high similarity to *Latilactobacillus graminis* (99.65%). The nucleotide sequences of strains BL1 and BL5 were registered in GenBank under accession numbers OP984707 and OP984708, respectively.

**Table 3:** The results of isolated lactic acid bacteria on the base of 16S rRNA gene sequences.

Strain	Accession number	16S rRNA gene sequencing data (closest relative)	Similarity (%)
BL1	NR_113335.1	*Limosilactobacillus fermentum* NBRC 15885	99.93
BL5	NR_042438.1	*Latilactobacillus graminis* G90(1)	99.65

### Characteristics of pre-ensiled native grass

The dry matter (DM) content of the three steppe types varied from 47.12–50.43% and CP content was 12.05% (MS), 10.90% (TS), and 12.45% (DS), respectively (**Table 4**). Relatively low WSC content (1.92-2.11%) and low LAB number (1.37-2.78 log cfu/g FM) were observed. No coliform bacteria were detected in the raw materials of MS and TS, and no mold was detected in the three steppe types.

**Table 4:** Chemical composition and microbial population of native grass prior to ensiling.

	Items	Types			SEM	*p*-value
		MS	TS	DS		
Chemical composition	DM (%FM)	47.12	49.65	50.43	1.00	0.1251
	OM (%DM)	92.94^b^	95.22^a^	91.51^c^	1.08	0.0006
	CP (%DM)	12.05	10.90	12.45	0.46	0.2192
	NDF (%DM)	63.86	66.99	65.58	0.91	0.1419
	ADF (%DM)	38.41^ab^	36.66^b^	41.05^a^	1.28	0.0213
	WSC (%DM)	1.92	2.11	2.05	0.06	0.1236
	EE (%DM)	3.49	3.29	3.52	0.07	0.2329
Microbial counts	Lactic acid bacteria (lg cfu/g FM)	1.37	2.63	2.78	0.45	0.3122
	Aerobic bacteria (lg cfu/g FM)	6.21	5.61	5.36	0.25	0.2309
	Coliform bacteria (lg cfu/g FM)	ND	ND	4.03	1.34	<0.0001
	Yeasts (lg cfu/g FM)	3.69^b^	4.14^ab^	4.40^a^	0.21	0.0520
	Mold (lg cfu/g FM)	ND	ND	ND	1.17	<0.0001

FM, fresh matter; DM, dry matter; CP, crude protein; NDF, neutral detergent fiber; ADF, acid detergent fiber; WSC, water-soluble carbohydrate; EE, ether extract. MS, meadow steppe; TS, typical steppe; DS desert steppe; ND not detected. Different lowercase letters indicate significant differences between treatments (*p* < 0.05); the same letters indicate non-significant (*p* > 0.05).

### Chemical characteristics of native grass silage

The silage of TS showed higher DM and organic matter (OM) contents than those of MS and DS in all treatments (*p* < 0.05), and TS silage showed higher NDF and WSC contents than MS and DS in CK and L5 treatment (**Table 5**). The EE contents showed no significant difference among the three steppe types (*p* > 0.05). The influence of LAB inoculants on the OM, CP, NDF and WSC of native grass was significant, except effect of LAB inoculants on CP and NDF in MS.

**Table 5:** Chemical compositions on 60 days of ensiling.

Items	Type	Treatment					Significance			
		CK	L1	L5	LP	LB	SEM	T	A	T*A
DM (%FM)	MS	35.28^C,bc^	35.95^C,ab^	34.50^C,c^	36.78^B,a^	36.28^C,ab^	1.32	<0.0001	0.3164	0.0003
	TS	47.04^A^	47.23^A^	48.00^A^	46.89^A^	47.04^A^				
	DS	40.97^B,a^	38.66^B,b^	38.66^B,b^	37.64^B,b^	37.78^B,b^				
OM (%DM)	MS	93.87^B,a^	93.93^B,a^	93.52^C,b^	93.93^B,a^	93.79^B,ab^	0.17	<0.0001	0.3999	0.0041
	TS	95.01^A,ab^	94.60^A,b^	94.95^A,ab^	94.67^A,ab^	95.04^A,a^				
	DS	93.53^B,ab^	93.71^B,a^	93.82^B,a^	93.27^C,ab^	93.02^B,b^				
CP (%DM)	MS	10.23	10.76^A^	10.69^A^	11.07^A^	10.10^A^	0.31	<0.0001	0.7190	0.0258
	TS	8.38^ab^	8.53^B,a^	8.66^B,a^	7.41^B,c^	7.63^B,bc^				
	DS	9.90^ab^	9.12^B,b^	9.98^A,ab^	10.65^A,a^	10.51^A,a^				
NDF (%DM)	MS	58.55^B^	55.21^B^	54.47^B^	56.57^A^	58.23^A^	1.01	<0.0001	0.6928	0.0512
	TS	61.23^A,ab^	61.77^A,a^	60.36^A,b^	60.37^A,b^	61.10^A,ab^				
	DS	52.06^C,ab^	55.15^B,a^	55.40^B,a^	50.90^B,b^	49.70^B,b^				
ADF (%DM)	MS	35.41	35.12^AB^	34.69^B^	34.55	35.78^A^	0.35	<0.0001	0.3204	0.3635
	TS	34.97^a^	33.36^B,bc^	32.53^C,c^	34.00^b^	32.87^B,c^				
	DS	36.58	36.03^A^	36.68^A^	36.37	36.18^A^				
WSC (%DM)	MS	0.76^B,b^	0.66^B,c^	0.84^B,a^	0.68^B,c^	0.72^bc^	0.02	<0.0001	<0.0001	0.8821
	TS	0.93^A,ab^	0.81^A,b^	0.99^A,a^	0.79^A,b^	0.84^ab^				
	DS	0.77^B,ab^	0.72^AB,b^	0.82^B,a^	0.72^B,b^	0.76^ab^				
EE (%DM)	MS	3.64	3.22	3.48	3.43	3.29	0.05	0.2374	0.2721	0.9890
	TS	3.59	3.28	3.53	3.75	3.47				
	DS	3.61	3.48	3.71	3.86	3.56				

FM, fresh matter; DM, dry matter; CP, crude protein; NDF, neutral detergent fiber; ADF, acid detergent fiber; WSC, water-soluble carbohydrate; EE, ether extract. CK, control group; L1, strain BL1 group; L5, strain BL5 group; LP, Lactiplantibacillus plantarum group; LB, Lentilactobacillus buchneri group. MS, meadow steppe; TS, typical steppe; DS desert steppe. T, steppe types; A, silage additives. Different capital letters indicate significant differences between steppe types and different lowercase letters indicate significant differences between treatments (*p* < 0.05); the same letters indicate non-significant (*p* > 0.05).

### Fermentation quality of native grass silage

Steppe types had significant effects on the pH (except L1) and NH_3_-N content of silage (*p* < 0.05) (**Table 6**). DS silage showed lower NH_3_-N content than MS and TS silages (*p* < 0.05). MS silage showed higher LA/AA than TS and DS in L5 treatment. LAB inoculants had significant effects on pH, PA, BA, NH_3_-N and LA/AA of silage (*p* < 0.05). Silage containing LAB inoculant showed significantly lower pH and BA content than CK (*p* < 0.05). L1 and LB treatments of the MS and TS showed higher AA content than other treatments (*p* < 0.05).

**Table 6:** Fermentation characteristics on 60 days of ensiling.

Items	Type	Treatment					Significance			
		CK	L1	L5	LP	LB	SEM	T	A	T*A
pH	MS	4.83^B,a^	4.29^cd^	4.27^A,d^	4.40^A,c^	4.65^A,b^	0.11	<0.0001	<0.0001	<0.0001
	TS	5.19^A,a^	4.28^b^	4.04^B,c^	4.15^B,bc^	4.16^C,bc^				
	DS	4.45^C,a^	4.21^c^	3.91^C,d^	4.32^A,b^	4.41^B,ab^				
LA (%FM)	MS	1.48	1.57	1.72^A^	1.66	1.50^A^	0.04	0.0012	0.0111	0.9567
	TS	1.32	1.33	1.40^B^	1.54	1.34^B^				
	DS	1.46^c^	1.54^abc^	1.75^A,ab^	1.77^a^	1.52^A,bc^				
AA (%FM)	MS	0.62^b^	0.98^a^	0.58^B,b^	0.61^B,b^	0.89^a^	0.04	0.0213	<0.0001	0.9131
	TS	0.71^b^	0.92^a^	0.74^A,b^	0.74^AB,b^	0.87^a^				
	DS	0.80	1.03	0.78^A^	0.78^A^	0.97				
PA (%FM)	MS	0.18^B^	0.11	0.05	0.14	0.00	0.03	0.0565	0.1432	0.3615
	TS	0.31^AB^	0.16	0.00	0.30	0.26				
	DS	0.43^A^	0.15	0.34	0.19	0.12				
BA (%FM)	MS	0.44^AB,a^	0.00^B,b^	0.00^b^	0.10^b^	0.00^b^	0.04	0.0225	<0.0001	0.0428
	TS	0.34^B,a^	0.20^A,b^	0.00^d^	0.17^bc^	0.10^c^				
	DS	0.57^A,a^	0.17^AB,b^	0.00^b^	0.13^b^	0.11^b^				
NH_3_-N(%TN)	MS	1.55^A,a^	1.22^A,ab^	1.37^A,ab^	1.17^A,b^	1.32^A,ab^	0.07	<0.0001	0.0118	0.4149
	TS	1.14^AB,a^	1.03^A,ab^	0.93^B,b^	1.02^A,ab^	1.02^B,ab^				
	DS	0.84^B,a^	0.81^B,a^	0.66^C,b^	0.67^B,b^	0.73^C,ab^				
LA/AA	MS	2.43^a^	1.66^b^	2.95^A,a^	2.74^a^	1.69^b^	0.12	0.0019	<0.0001	0.6346
	TS	1.92^ab^	1.44^b^	1.91^B,ab^	2.10^a^	1.54^ab^				
	DS	2.03^ab^	1.51^b^	2.23^B,ab^	2.29^a^	1.57^ab^				

FM, fresh matter; LA, lactic acid; AA, acetic acid; PA, propionic acid; BA, butyric acid; NH_3_-N, ammonia nitrogen; TN, total nitrogen. CK, control group; L1, strain BL1 group; L5, strain BL5 group; LP, Lactiplantibacillus plantarum group; LB, Lentilactobacillus buchneri group. MS, meadow steppe; TS, typical steppe; DS desert steppe. T, steppe types; A, silage additives. Different capital letters indicate significant differences between steppe types and different lowercase letters indicate significant differences between treatments (*p* < 0.05); the same letters indicate non-significant (*p* > 0.05).

### Microbial population of native grass silage

Steppe types significantly influenced LAB counts in CK and L1 treatment (*p* < 0.05), and significantly influenced yeast counts in LP and LB treatment (*p* < 0.05), but had no significant effect on aerobic bacteria counts (*p* > 0.05) (**Table 7**). Silages of MS and TS showed higher LAB counts than DS in CK treatment. LAB inoculants significantly influenced the number of LAB in MS and DS (*p* < 0.05), and significantly influenced aerobic bacteria counts in MS (*p* < 0.05) but had no significant effect on yeast counts (*p* > 0.05). Additive treatments showed higher LAB counts than CK in MS and DS, and LB silages in these two steppe native grass had higher LAB counts compared with other treatments (*p* < 0.05). The LP treatment of MS silage had higher aerobic bacteria counts than the other treatments (*p* < 0.05). Mold and coliform bacteria were not found in the different treatments.

**Table 7:** Microbial populations on 60 days of ensiling.

Items	Type	Treatment					Significance			
		CK	L1	L5	LP	LB	SEM	T	A	T*A
LAB (lg cfu/g FM)	MS	7.37^A,c^	7.74^A,ab^	7.44^bc^	7.50^bc^	8.03^a^	0.10	0.0002	0.0003	0.2855
	TS	7.12^A^	7.32^AB^	7.48	7.65	7.60				
	DS	6.59^B,c^	6.82^B,bc^	6.92^bc^	7.41^ab^	7.65^a^				
Yeasts (lg cfu/g FM)	MS	6.04	5.81	5.60	6.37^A^	5.95^AB^	0.12	0.0007	0.7672	0.7084
	TS	5.30	5.29	5.22	5.52^B^	5.34^B^				
	DS	6.54	6.47	6.05	5.76^AB^	6.52^A^				
Aerobic bacteria (lg cfu/g FM)	MS	6.55^ab^	6.02^c^	6.07^bc^	6.90^a^	6.23^bc^	0.10	0.4773	0.0359	0.9021
	TS	6.26	6.13	5.89	6.51	6.19				
	DS	6.93	5.88	5.96	6.76	6.77				
Coliform bacteria (lg cfu/g FM)	MS	ND	ND	ND	ND	ND	0.00	——	——	——
	TS	ND	ND	ND	ND	ND				
	DS	ND	ND	ND	ND	ND				
Molds (lg cfu/g FM)	MS	ND	ND	ND	ND	ND	0.00	——	——	——
	TS	ND	ND	ND	ND	ND				
	DS	ND	ND	ND	ND	ND				

FM, fresh matter; LAB, lactic acid bacteria. CK, control group; L1, strain BL1 group; L5, strain BL5 group; LP, Lactiplantibacillus plantarum group; LB, Lentilactobacillus buchneri group. MS, meadow steppe; TS, typical steppe; DS desert steppe. T, steppe types; A, silage additives. Different capital letters indicate significant differences between steppe types and different lowercase letters indicate significant differences between treatments (*p* < 0.05); the same letters indicate non-significant (*p* > 0.05).

### Bacterial community of native grass silage

The 16S rDNA sequences were subjected to high-throughput sequencing to calculate and assess bacterial diversity in the silage of different steppe types (**Table 8**). The coverage depth for all treatments was above 96%. The ACE index was significantly higher for L1 than FM and LP in silage from MS (*p* < 0.05), however, the ACE and Chao1 index was significantly higher for L5 than L1 and LB in silage from TS (*p* < 0.05). The Simpson index of L1 was significantly lower than FM, CK, L5 and LP (*p* < 0.05) and the Shannon index was significantly lower than FM, CK and L5 (*p* < 0.05) in silage from MS. The Simpson and Shannon indices were significantly higher (*p* < 0.05) in TS silage for CK and L5 than for the other treatments. The Simpson and Shannon indices were significantly higher (*p* < 0.05) but Coverage was significantly lower (*p* < 0.05) for FM than for the other treatments in the DS silage. In addition, the Simpson and Shannon indices differed between treatments for the three steppe types of natural forage, including FM, with the exception of LB (*p* < 0.05).

**Table 8:** Alpha-diversity of bacterial diversity of native grass silages of different steppe types.

Items	Type	Treatment						Significance			
		FM	CK	L1	L5	LP	LB	SEM	T	A	T*A
Ace	MS	68.74^b^	106.29^ab^	212.89^a^	91.94^ab^	71.98^b^	96.63^ab^	9.29	0.0607	0.6944	0.1908
	TS	64.12^abc^	73.12^ab^	41.92^c^	80.97^a^	57.29^abc^	47.07^bc^				
	DS	75.75	60.16	76.95	55.34	100.85	134.23				
Chao1	MS	69.30	68.44	77.87	80.29	52.00	60.67	3.02	0.1695	0.6842	0.5515
	TS	52.58^ab^	71.06^a^	35.89^b^	76.80^a^	48.82^ab^	40.71^b^				
	DS	55.17	58.55	52.78	46.87	58.42	66.83				
Simpson	MS	0.39^B,a^	0.36^B,a^	0.11^AB,b^	0.47^A,a^	0.35^A,a^	0.27^ab^	0.05	0.3028	<0.0001	<0.0001
	TS	0.21^B,b^	0.66^A,a^	0.25^A,b^	0.76^A,a^	0.14^B,b^	0.24^b^				
	DS	0.74^A,a^	0.29^B,cd^	0.06^B,e^	0.11^B,de^	0.50^A,b^	0.37^bc^				
Shannon	MS	1.66^B,a^	1.27^B,a^	0.48^AB,b^	1.65^A,a^	1.01^AB,ab^	0.99^ab^	0.19	0.4280	<0.0001	<0.0001
	TS	0.69^B,b^	2.58^A,a^	0.79^A,b^	2.78^A,a^	0.55^B,b^	0.68^b^				
	DS	2.91^A,a^	1.08^B,bc^	0.29^B,c^	0.48^B,c^	1.45^A,b^	0.97^bc^				
Coverage	MS	0.9963	0.9966	0.9960	0.9948	0.9970	0.9972	0.00	0.0723	0.0188	0.0055
	TS	0.9975	0.9967	0.9981	0.9962	0.9981	0.9984				
	DS	0.9608^b^	0.9976^a^	0.9979^a^	0.9978^a^	0.9954^a^	0.9975^a^				

FM, fresh material; CK, control group; L1, strain BL1 group; L5, strain BL5 group; LP, Lactiplantibacillus plantarum group; LB, Lentilactobacillus buchneri group. MS, meadow steppe; TS, typical steppe; DS desert steppe. T, steppe types; A, silage additives. Different capital letters indicate significant differences between steppe types and different lowercase letters indicate significant differences between treatments (*p* < 0.05); the same letters indicate non-significant (*p* > 0.05).

**Figure 1:**
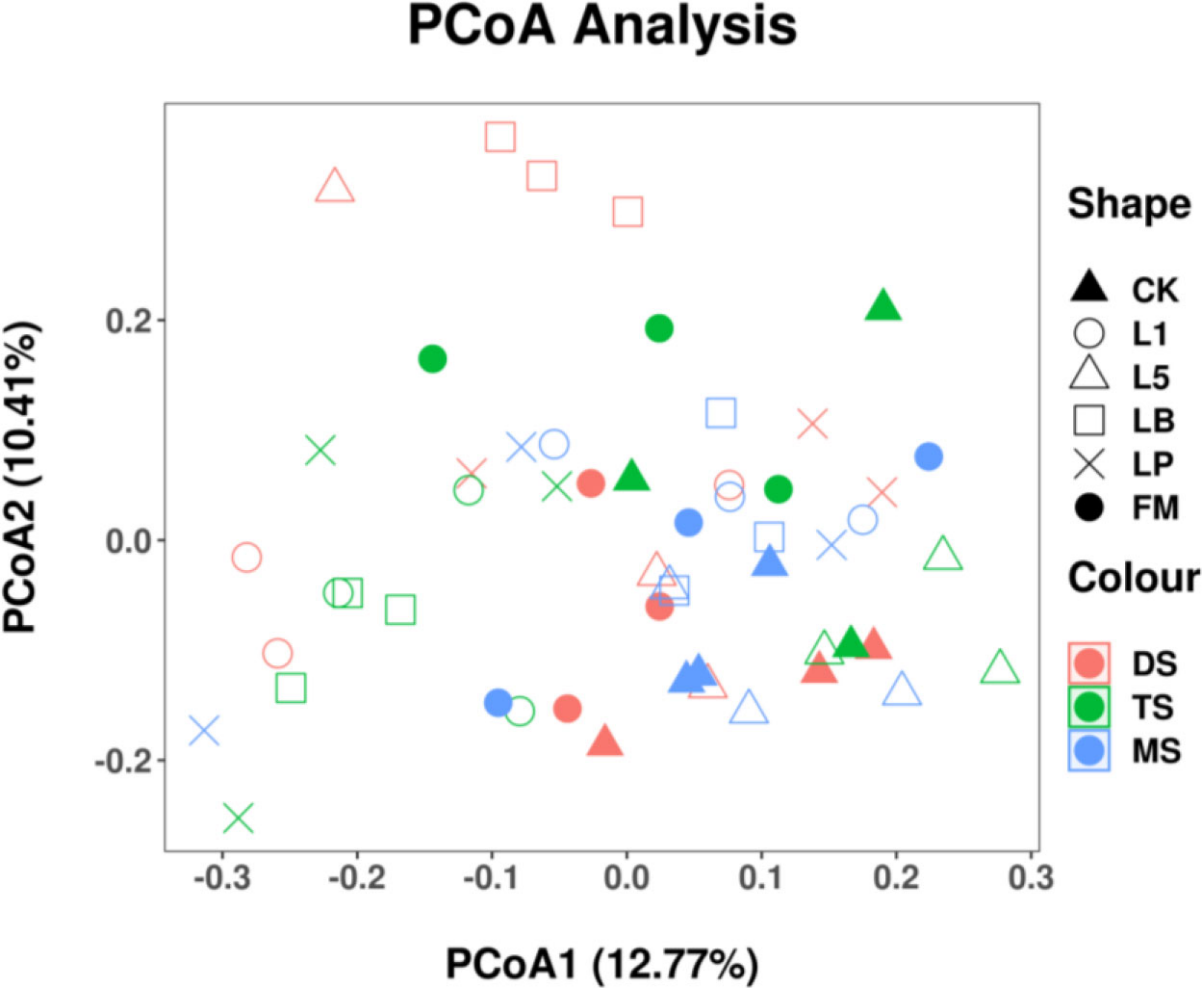
Principal coordinate analysis (PCoA) of the bacterial community of fresh material and native grass on 60 days of ensiling. FM, fresh material; CK, control group; L1, strain BL1 group; L5, strain BL5 group; LP, *Lactiplantibacillus plantarum* group; LB, *Lentilactobacillus buchneri* group. MS, meadow steppe; TS, typical steppe; DS desert steppe.

To determine whether bacterial community structure differed between the raw material and silage of different steppe types, principal coordinate analysis (PCoA) was performed based on Unifrac (unweighted) distances (**Figure 1**). The bacterial communities of raw materials from different steppe types were distinct. Distance was also observed between the bacterial communities of CK and LAB treatments.

**Figure 2:**
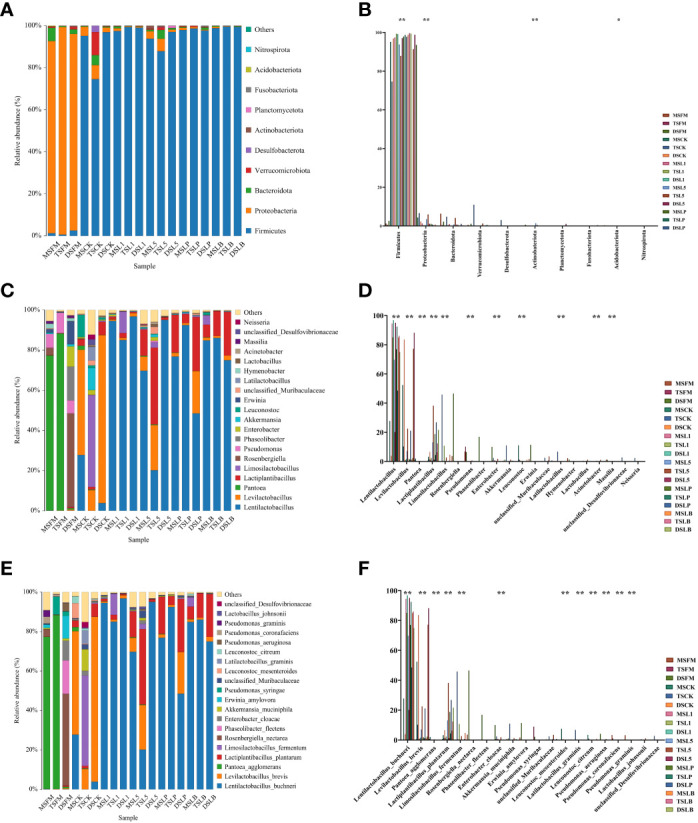
The bacterial community of fresh material and native grass on 60 days of ensiling of different steppe types. **(A)** The bacterial community was shown at the phylum level. **(B)** The extended error bar plot displaying the significant differences among groups at the phylum level. **(C)** The bacterial community was shown at the genus level. **(D)** The extended error bar plot displaying significant differences among groups at the genus level. **(E)** The bacterial community was shown at the genus level. **(F)** The extended error bar plot displaying significant differences among groups at the genus level. *Shows that the significant difference was at *p* < 0.05 level. **Shows that the significant difference was at *p* < 0.01 level. FM, fresh material; CK, control group; L1, strain BL1 group; L5, strain BL5 group; LP, *Lactiplantibacillus plantarum* group; LB, *Lentilactobacillus buchneri* group. MS, meadow steppe; TS, typical steppe; DS desert steppe.

The predominant phylum in the three native grass steppe samples was Proteobacteria (>90%) (**Figure 2A**). However, their community composition was influenced by ensiling. By the end of the fermentation process, Firmicutes prevailed in all silage samples of the three steppe types, accounting for more than 87% in all groups (except the TSCK treatment). To further reveal phylum-level differences in the bacterial communities of native grass silage based on steppe types and LAB inoculants, one-way analysis of variance (ANOVA) was conducted on the community composition of native grasses before and after ensiling (**Figure 2B**). Significant differences were observed in the abundance of Acidobacteriota (*p* < 0.05), and highly significant differences were detected in the abundance of Firmicutes, Proteobacteria, and Actinobacteriota (*p* < 0.01), which were attached to fresh and silage samples of native grass in different treatments.

The relative abundance of bacterial communities in the pre-ensiled native grass was very low (**Figure 2C**). The bacterial composition of FM at the genus level differed among the three steppe types. The main epiphytic bacteria associated with the FM of different steppe types were *Pantoea* in MS and TS, and *Rosenbergiella* and *Phaseolibacter* in DS, although the abundance of these genera decreased after ensiling. After 60 days of fermentation, *Levilactobacillus* was the most common genus in the CK treatments of MS and DS, and *Limosilactobacillus* was the main genus in the CK treatment of TS. Compared to CK, with the exception of TSL5, all treatments had *Levilactobacillus* as the dominant genus for all three native grass steppe types, and TSL5 was dominated by the *Lactiplantibacillus*. The abundance of *Lentilactobacillus*, *Levilactobacillus*, *Pantoea*, *Lactiplantibacillus*, *Limosilactobacillus*, *Pseudomonas*, *Enterobacter*, *Leuconostoc*, *Latilactobacillus*, *Acinetobacter*, and *Massilia* showed highly significant differences (*p* < 0.01) (**Figure 2D**).

The main epiphytic bacteria associated with the FM of MS and TS were *Pantoea agglomerans*, and those associated with the FM of DS were *Rosenbergiella nectarea* and *Phaseolibacter flectens*, although the abundance of these species decreased after ensiling (**Figure 2E**). After 60 days of fermentation, *Levilactobacillus brevis* was the most common species in the CK treatment of MS and DS, while *L. fermentum* was the main species in the CK treatment of TS. Compared with CK, all LAB silage treatments showed *L. brevis*, *L. fermentum*, and *L. plantarum* as the main species (>80% abundance). The abundance of *L. buchneri*, *L. brevis*, *Pantoea agglomerans*, *L. plantarum*, *L. fermentum*, *Enterobacter cloacae*, *Leuconostoc mesenteroides*, *L. graminis*, *Leuconostoc citreum*, *Pseudomonas aeruginosa*, *Pseudomonas coronafaciens*, and *Pseudomonas graminis* showed highly significant differences (*p* < 0.01) (**Figure 2F**).

**Figure 3:**
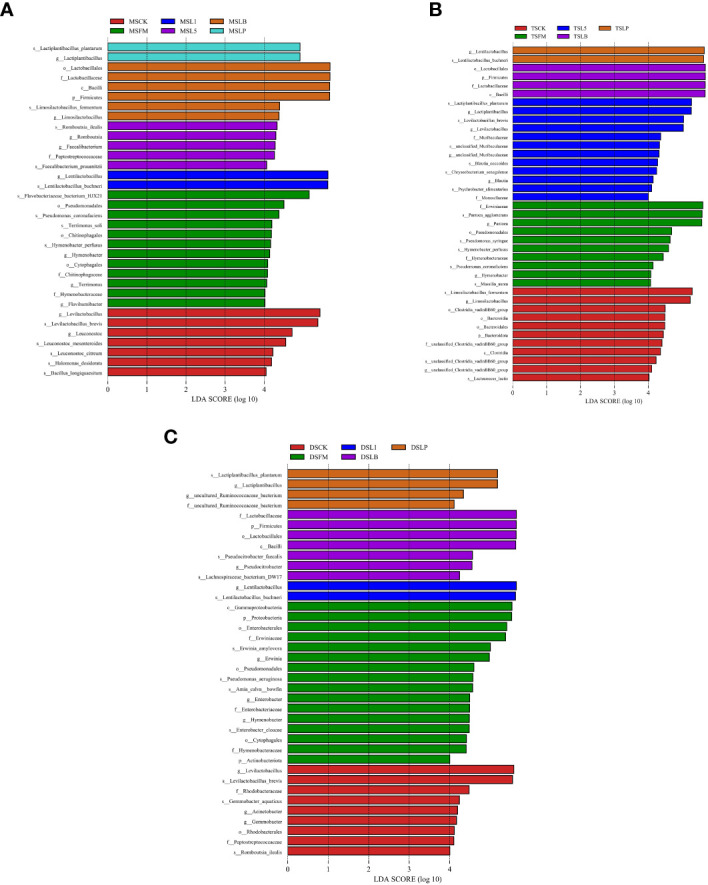
The linear discrimination analysis (LDA) coupled on the bacterial community of fresh material and native grass on 60 days of ensiling, with effect size (LEfSe) analysis. The significant difference in species was estimated by an LDA score greater at default score = 4.0. The length of the histogram shows the LDA score of differences in these groups. FM, fresh material; CK, control group; L1, strain BL1 group; L5, strain BL5 group; LP, *Lactiplantibacillus plantarum* group; LB, *Lentilactobacillus buchneri* group. MS, meadow steppe; TS, typical steppe; DS desert steppe. **(A)** LEfSe analysis between different treatment groups in meadow steppe (MS). **(B)** LEfSe analysis between different treatment groups in typical steppe (TS). **(C)** LEfSe analysis between different treatment groups in desert steppe (DS).

The LEfSe method was used to evaluate differences in microbial communities of the three steppe types and to explore specific bacterial species in each group (LDA score > 4.0). Steppe type and LAB inoculant had a tremendous influence on the resulting bacterial community (**Figure 3**). In MS samples, 34 bacteria were significantly enriched, and *Lactobacillaceae* showed the highest LDA score (5.68) (**Figure 3A**). In TS samples, 39 bacteria were significantly enriched, and *Lactobacillaceae* showed the highest LDA score (5.68) (**Figure 3B**). In DS samples, 38 bacteria were significantly enriched, and *Lactobacillaceae* showed the highest LDA score (5.64) (**Figure 3C**).

**Figure 4:**
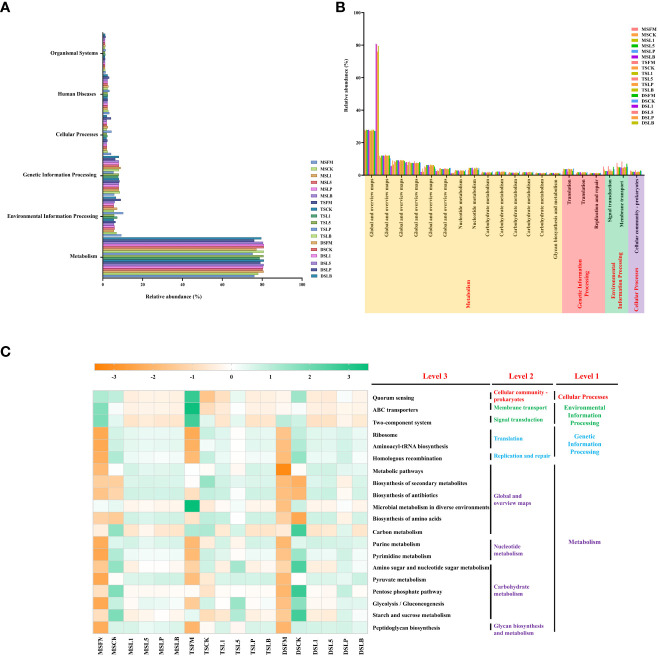
Functional predictions for silage microbiota with significantly different KEGG pathways (*p <*0.05) among the eighteen groups of silage. KEGG pathways at Level 1 **(A)**, Level 2 **(B)**, and Level 3 **(C)** are represented. FM, fresh material; CK, control group; L1, strain BL1 group; L5, strain BL5 group; LP, *Lactiplantibacillus plantarum* group; LB, *Lentilactobacillus buchneri* group. MS, meadow steppe; TS, typical steppe; DS desert steppe.

PICRUSt predicts the bacterial metabolic function in accordance with the KEGG pathway. In total, six different metabolic pathways were identified in the raw materials and silages of the three native grass steppe types (**Figure 4A**). Among them, metabolism metabolic abundance accounted for the largest proportion (>75% abundance), followed by environmental information processing and genetic information processing. The top 20 metabolic functions in level 2 had 14 metabolic pathways assigned to metabolism, 3 metabolic pathways assigned to genetic information processing (**Figure 4B**). There were 2 and 1 metabolic pathways assigned to environmental information processing and cellular processing, respectively. The amino sugar and nucleotide sugar metabolism, pyruvate metabolism, and glycolysis/gluconeogenesis pathways were significantly enriched in the three steppe types after ensiling (level 3), while pentose phosphate pathway and starch and sucrose metabolism were enriched in MS and DS treatments (**Figure 4C**). In terms of carbohydrate metabolism, the abundance of amino sugar and nucleotide metabolism and pentose phosphate pathway, glycolysis/glycoprotein production, and starch and sucrose metabolism were higher in the CK treatment than in the LAB additive treatments from MS and DS, while the abundance of amino sugar and nucleotide sugar metabolism, glycolysis/gluconeogenesis, and starch and sucrose metabolism was higher in the L5 treatment than in other treatments in TS. In terms of global and overview maps, the abundance of metabolic pathways, biosynthesis of secondary metabolites, biosynthesis of antibiotics, biosynthesis of amino acids, and carbon metabolism was enriched after ensiling. Notably, the abundance of microbial metabolism in diverse environments and carbon metabolism was significantly reduced after ensiling.

## Discussion

LAB have been found and identified in forage silage, fermented food, and dairy products. In addition, LAB have been shown to be the dominant bacteria in various forage and silage samples (Burns et al., 2018). Nonetheless, it can be a challenge to identify the differences among dichotomous species based on morphological, physiological, and biochemical tests (Wang et al., 2019a). Therefore, this study combined with actual silage application to further evaluate and screen the selected LAB. The 16S rDNA sequence analysis has widely used to identify organisms at the genus and species levels (Wang et al., 2019b).

Chen et al. (2017) and Chu-Ky et al. (2014) concluded that *L. fermentum* is highly tolerant to acidity and can grow in low pH environments, which is consistent with the results of this study. Both strains differed not only in their ability to grow in an acidic environment but also in the temperature of the growth environment, which indicates that different LAB differ in their adaptability to the environment in which they grow (Du et al., 2022). In addition, *L. fermentum* was able to generate more fermentation products compared with strain BL5.

In this study, the DM and WSC contents of native grass was comparatively high, equivalent to that in a dry environment, which could be explained by the different plant species was observed in the three steppes and could support effective acid production for bacteria (Nkosi et al., 2016). A wide range of bacteria attached to the plant surface and different silage materials cultivate different microorganisms, in terms of both composition and population (Khota et al., 2016). Previously published study indicated that the ratio of beneficial to undesirable microorganism was estimated at 1:10 (Lin et al., 1992). In this study, the counts of undesirable microorganism, such as mold and coliform, were lower than the detectable levels in all steppe types, excepted the DS. These results could be included by the compositions of the steppes (Du et al., 2022). In the current study, the numbers of LAB on plants were lower than 5.0 log_10_ cfu/g FM, which is in accordance with the previous reports (Pang et al., 2012; Du et al., 2022). Therefore, it was necessary to isolate and selecte LAB additives for these steppes.

In the present study, the fermentation characteristics of the three steppe types changed after ensiling, probably because of the complex plant community structure of the native grasslands. Notably, the DM and CP content among these treatments was similar. This higher LA content in these treatments limited the microorganism that could breakdown the DM and CP contents might be the main reason (Muck, 1988; Muck, 2010). In addition, the lack of sufficient substrates for native grass of different steppe types to inhibit protease activity is another reason for this result (Sousa et al., 2019). The NDF and ADF contents showed no significant difference between CK and the four additive treatments, because the majority of LAB additives did not have the ability to produce cellulase and hemi-cellulase to degrade forage cell walls (Xu et al., 2020).

The organic acid (mainly LA and AA) and NH_3_-N contents, and pH are important indicators for well-preserved forage silage (Hashemzadeh-Cigari et al., 2014). In the high DM silage such as straw forage and native grass, the water activity of LAB is insufficient, so the silage fermentation is reduced due to insufficient metabolic water required for the growth of LAB (Borreani et al., 2018). Four LAB strains produced a large amount of LA and a certain amount of AA to decrease the pH and inhibit the activity of proteases in plants and microbes and reduce proteolysis using a series of substrates (Dong et al., 2022; Lu et al., 2022). Similarly, Si et al. (2019) proved that the homo- and hetero- fermentative LAB inoculants could decrease the NH_3_-N content and pH during ensiling.

Sequencing of bacteria in fresh native grass and silage samples was carried out by amplicon sequencing. The coverage values for all samples were approximately 0.96. This indicated that the sequencing depth was quite extensive, which justified the probing of characteristics of the bacterial microbial community (Keshri et al., 2019). It is generally believed that these specific operational taxonomic units (OTUs) might be responsible for the differences in silage quality (Yang et al., 2019). In a recent study, a significant reduction in bacterial diversity and species richness was observed as the complex microbial community of FM was eventually replaced by LAB, which dominated the entire fermentation process (Cui et al., 2022). The higher Simpson and Shannon indices for MSL5 and TSL5 and the lower levels exhibited by DSL1 may be due to the higher number and relative abundance of bacteria dominating the fermentation in MSL5 and TSL5, as indicated by the bacterial community composition.

The PCoA plots revealed a clear separation of bacteria in the raw materials and silages of different steppe types, indicating that the bacterial community is altered by the silage technology. The PCoA of silages inoculated with LAB were also separated from the control, which demonstrated a significant effect of the additive on the bacterial community. These results were in agreement with the previous study that reported by Zeng et al. (2020). Therefore, based on the results of α-diversity and β-diversity, the LAB inoculations could influence the microbial diversity and community structure of native grass silage.

As shown, the epiphytic microorganisms could regulates the rate and extent of fermentation. Conversely, the microbial community is formed by the fermentation products. Zhao et al. (2017) concluded that proteobacteria showed the highest relative abundance in the bacterial community of pasture silage feedstock, which is consistent with the results of this study. At the phylum level, the relative abundance of Firmicutes in silage with or without additives increased significantly to become the most important phylum compared to raw materials, while the relative abundance of Proteobacteria decreased significantly, indicating that anaerobic conditions and exogenous additives effectively promote effective acid production by beneficial bacteria in silage and create an acidic environment that inhibits the reproductive activity of spoilage microorganisms (McGarvey et al., 2013).

At the genus level, the dominant epiphytes in fresh native grass were *Pantoea* and *Rosenbergiella*, which were similar to the dominant epiphytes in fresh alfalfa (Ogunade et al., 2018) but different from the dominant epiphytes in fresh *E. nutans* (Ding et al., 2020). It has been suggested that altitude, plant composition and climate can influence the distribution of LAB (Leong et al., 2014). After fermentation process, the portions of *Pantoea* and *Rosenbergiella* decreased significantly and were replaced by *Lentilactobacillus*, *Levilactobacillus*, *Lactiplantibacillus*, and *Limosilactobacillus* in different LAB inoculant treatments, which is in agreement with the results of You et al. (2022). The high sensitivity of *Pantoea, Rosenbergiella*, and *Lactobacillus* to the changes in pH may be the main reasons (Li et al., 2022). Furthermore, the abundance of *Lentilactobacillus* and *Lactiplantibacillus* increased in four LAB inoculant treatments compared to the CK, indicating that the inoculated strains were not competed out by the microbial populations attached to the plants. This was probably because the inoculated strains dominated and controlled the microbial community during the fermentation process (Wang et al., 2006).

There were significant species-level differences in epiphytic bacteria among the FMs of different steppe types. The dominant species in the raw material of DS was *Rosenbergiella nectarea*, and the dominant species in both TS and MS was *Pantoea agglomerans*. This result was different from the results of previous studies (Bao et al., 2016; Guan et al., 2018; Li et al., 2021). The dominant species in the CK treatment of the three steppe types were *Levilactobacillus brevis* and *L. fermentum*, which confirmed that harmful epiphytic bacteria in FM were rapidly inhibited under anaerobic environment and low pH stress. There were also some differences in silage containing different LAB species. *L. buchneri* was the dominant species in BL1 and BL5 treatments. Notably, *L. fermentum* and *L. graminis* were not detected among the L1- and L5-attached isolates, mainly because both bacterial species were at the early stages of fermentation and were not present in sufficient numbers to be detected at the late stages of silage fermentation (Beck et al., 1988; Meenakshi Malhotra, 2015). *L. plantarum* and *L. buchneri* were the dominant species in LP and LB treatments. This is mainly because, as the fermentation process continues, the temperature of silage sample increases, which usually leads to the transformation of LAB from the homofermentative to the heterofermentative type.

Functional KEGG profiles of bacterial communities were inferred using 16S rRNA gene sequences. The carbohydrate metabolic pathway was enriched after ensiling, which may be because of the fermentation of available carbohydrates into energy and short-chain fatty acids during ensiling (Zhao et al., 2022). The pyruvate metabolism, pentose phosphate pathway, and glycolysis/gluconeogenesis metabolic pathway dominated carbohydrate metabolism in native grass silage treatment of the three steppe types. Du et al. (2022) reported metabolic pathways and their influence on the taste (sweet and sour) and palatability of native grass silage, which is similar to the findings of this study. The enrichment of pyruvate metabolism showed no difference among the silages containing LAB additives. By contrast, the enrichment of pentose phosphate pathway, glycolysis/gluconeogenesis, starch and sucrose metabolism, and amino sugar and nucleotide sugar metabolism increased in the DSLP and TSL5 treatments compared with the other treatments, possibly because of the higher abundance of *L. plantarum* and *Levilactobacillus brevis* in these two treatments (Su et al., 2021). Thus, there were showed different abundance in the level 3 of metabolism of carbohydrate metabolism (**Table 5**). Additionally, the enrichment of starch and sucrose metabolism in the five silage treatments compared with the FM showed different advantages, indicating that the epiphytic LAB utilized the WSC to different degrees in the silage. The enrichment of amino acid metabolism decreased in the TSL5 and DSLP treatments compared with the other treatments. This may be because of two reasons: 1) high CP content (most of the protein is not broken down into NH_3_-N during fermentation), and 2) higher abundance of *L. plantarum*, which can promptly decrease pH during the early stages of fermentation and then inhibit amino acid metabolism (Flythe and Russell, 2004).

In summary, bacterial communities of fresh samples of different steppe types of native grasses were found to be dominated by *Pantoea agglomerans*. The bacterial communities of different steppe types of native grasses with and without the addition of LAB changed significantly after fermentation. The fermentation characteristics of silages supplemented with self-screening strains (*L. fermentum* and *L. graminis*) and commercial LAB additives differed considerably due to their different fermentation pathways. The addition of *L. fermentum* and *L. graminis* increased the relative abundance of *L. buchneri* and *L. plantarum*, reduced the pH and rapidly inhibited undesirable microorganisms. Thus, *L. fermentum* and *L. graminis* showed potential to improve the fermentation of native grasses from different steppe types, providing the feasibility of new strains to improve the fermentation of native grass silage by regulating the epiphytic flora in silage to promote the fermentation of LA and AA, improving the fermentation characteristics and metabolic capacity of the microbiota.

## Data availability statement

The datasets presented in this study can be found in online repositories. The names of the repository/repositories and accession number(s) can be found below: https://www.ncbi.nlm.nih.gov/genbank/, PRJNA912573.

## Author contributions

JB: Conceptualization, Investigation, Visualization, Methodology, Formal analysis, Writing -original draft. GG: Conceptualization, Investigation, Formal analysis. ZW: Supervision, Writing-review and editing. YX: Supervision, Writing-review and editing. MZ: Conceptualization, Investigation, Formal analysis. LS: Conceptualization, Investigation, Formal analysis. YW: Conceptualization, Investigation, Formal analysis. JZ: Conceptualization, Investigation, Formal analysis. YJ: Conceptualization, Methodology, Validation, Investigation, Writing-Review and editing, Funding acquisition. SD: Conceptualization, Methodology, Validation, Investigation, Writing-Review and editing. All authors contributed to the article and approved the submitted version.
